# Correlation between pulmonary to systemic flow ratio and N-terminal Pro-B-type natriuretic peptide level in children with atrial septal defect

**DOI:** 10.3389/fcvm.2025.1522603

**Published:** 2025-03-14

**Authors:** Li-Chin Liao, Yun-Yu Chen, Yun-Ching Fu, Hui-Chih Hung

**Affiliations:** ^1^ Doctoral Program in Translational Medicine, National Chung Hsing University, Taichung, Taiwan; ^2^ Rong Hsing Translational Medicine Research Center, National Chung Hsing University, Taichung, Taiwan; ^3^ Department of Pediatrics, Wuri Lin Shin Hospital, Taichung, Taiwan; ^4^ Department of Pediatric Cardiology, Children’s Medical Center, Taichung Veterans General Hospital, Taichung, Taiwan; ^5^ Department of Life Sciences, National Chung Hsing University, Taichung, Taiwan; ^6^ Department of Medical Research, Taichung Veterans General Hospital, Taichung, Taiwan; ^7^ Cardiovascular Center, Taichung Veterans General Hospital, Taichung, Taiwan; ^8^ Heart Rhythm Center, Division of Cardiology, Department of Medicine, Taipei Veterans General Hospital, Taipei, Taiwan; ^9^ Cardiovascular Research Center, College of Medicine, National Chung Hsing University, Taichung, Taiwan; ^10^ Department of Pediatrics, School of Medicine, National Chung Hsing University, Taichung, Taiwan; ^11^ Department of Pediatrics and Institute of Clinical Medicine, National Yang Ming Chiao Tung University, Taipei, Taiwan; ^12^ iEGG & Animal Biotechnology Center, National Chung Hsing University, Taichung, Taiwan

**Keywords:** atrial septal defect, N-terminal pro-B-type natriuretic peptide, pulmonary to systemic flow ratio, children, intervention

## Abstract

**Introduction:**

Atrial septal defect (ASD) increases pulmonary to systemic flow ratio (*Q*_p_/*Q*_s_) which is an important determinant factor for treatment. N-terminal pro-B-type natriuretic peptide (NT-proBNP) levels are correlated with volume overloading of the heart. This study aims to explore the relationship between *Q*_p_/*Q*_s_ and NT-proBNP levels in children with ASD.

**Materials and methods:**

Between January 2010 and December 2023, 464 patients under 20 years old with ASD who underwent cardiac catheterization and received NT-proBNP test were enrolled retrospectively. Baseline characteristics such as sex, body weight, and age were recorded. *Q*_p_/*Q*_s_ was measured during standardized right heart catheterization according to Fick principle.

**Results:**

A significant positive correlation existed between NT-proBNP and *Q*_p_/*Q*_s_ (R = 0.507, *P* < 0.001), with an *R*^2^ of 0.258. The linear regression model indicates that a one-unit (pg/ml) increase in NT-proBNP corresponded to a 0.003-unit increase in *Q*_p_/*Q*_s_ (*P* < 0.001). Patients with a *Q*_p_/*Q*_s_ ratio ≥ 2 had significantly higher NT-proBNP levels than those with a ratio <2 (*P* < 0.001).

**Conclustion:**

This study, the largest cohort to date, reveals the correlation between non-invasive NT-proBNP level and invasive *Q*_p_/*Q*_s_ measurement in children with ASD.

## Introduction

Atrial septal defects (ASDs) represent around 10–15 percent of all congenital heart diseases. The estimated occurrence at birth is approximately 1–2 per 1,000 live births (1–4). The left-to-right shunt in ASD increases pulmonary to systemic flow ratio (*Q*_p_/*Q*_s_) ratio, leading to hemodynamic changes by increasing the volume load on the right heart and affecting the left heart (5–7). The indication for surgical repair is a *Q*_p_/*Q*_s_ greater than 2.0, as this imposes a significant burden on the heart (8, 9). N-terminal pro-B-type natriuretic peptide (NT-proBNP) is a hormone secreted by the heart in response to elevated pressure, volume overload, or cardiac stress. Previous studies have shown a correlation between NT-proBNP levels and volume overloading of the heart. However, there were limited studies with small sample sizes that explore the relationship between *Q*_p_/*Q*_s_ ratio and NT-proBNP levels in patients with ASD (10–12). Early intervention has the potential to reduce morbidity and mortality in children (7, 13, 14). However, Accurate measurement of *Q*_p_/*Q*_s_ typically requires cardiac catheterization, which is an invasive procedure. In contrast, NT-proBNP levels can be determined quickly and non-invasively through a simple blood test. Our aim is to explore the correlation between *Q*_p_/*Q*_s_ and NT-proBNP levels. If such a correlation exists, NT-proBNP could potentially serve as a non-invasive predictor of the hemodynamic burden in patients with ASD.

## Materials and methods

### Study participants

Between January 2010 and December 2023, 464 patients under 20 years old with ASD who underwent cardiac catheterization and received NT-proBNP test were enrolled retrospectively. The exclusion criteria were those who had congenital heart disease other than ASD, and patients who were not suitable for cardiac catheterization. This study was approved by the Committee on Human Studies (Institutional Review Board) at Taichung Veterans General Hospital (TCVGH-IRB no. CG16272B).

### Baseline data collection and hemodynamic assessment procedures

Baseline characteristics were collected including sex, body weight, age, and body surface area. All patients had no other congenital heart or systemic disease. All patients underwent right cardiac catheterization under conscious sedation. Hemodynamic parameters, such as pulmonary blood flow (*Q*_p_) and systemic blood flow (*Q*_s_), were calculated using the Fick formula. A normal *Q*_p_/*Q*_s_ ratio is 1, with a ratio exceeding 2 typically indicating a significant left-to-right shunt and volume overload. Pulmonary hypertension (PH) is diagnosed when the mean pulmonary artery pressure exceeds 20 mmHg, as measured directly in the cardiac catheterization lab. Venous blood was collected without fasting beforehand and 5–10 ml blood was stored in a tube without anticoagulant after admission and was transferred immediately to the hospital's Department of Laboratory Medicine. The normal reference range of NTproBNP was 0–125 pg/ml in our laboratory. The defect size was measured using standard echocardiographic techniques, including subcostal, precordial, or apical imaging over at least two cardiac cycles, with the results averaged.

### Statistical methods

Continuous variables were presented as mean ± standard deviation (SD), and categorical variables were summarized as absolute numbers and percentages. Group comparisons for continuous variables were conducted using Student's *t*-test for two groups, while categorical variables were analyzed using the Chi-square test. Linear regression was employed to assess the relationship between NT-proBNP (independent variable) and *Q*_p_/*Q*_s_ (dependent variable), with the strength of the association quantified by the correlation coefficient (*R*) and the coefficient of determination (*R*^2^). To further evaluate the effect of *Q*_p_/*Q*_s_ groups on NT-proBNP levels after multivariable adjustment, a generalized estimating equations (GEE) model with a linear link function was utilized. The multivariable model was adjusted for age, sex, body surface area, mean right atrium pressure (RAm), mean pulmonary artery pressure (PAm), fluoroscopy time, and procedure time. The results from the GEE analysis were reported as beta coefficients with 95% confidence intervals (CIs). Data were analyzed using SPSS Statistics (Version 23.0, Chicago, IL, USA). A *p*-value <0.05 was considered significant.

## Results

The baseline characteristics of the 464 patients with ASD enrolled in this study are presented in Table 1. The cohort consisted of 200 males (43.1%) and 264 females (56.9%) with a mean age of 7.8 ± 4.7 years. The mean body weight was 27.4 ± 16.6 kg. Hemodynamic assessment via right heart catheterization revealed a RAm of 5.3 ± 3.3 mmHg and a PAm of 17.3 ± 4.9 mmHg. The mean *Q*_p_/*Q*_s_ ratio was 1.89 ± 0.67, with 130 patients (28.0%) presenting a *Q*_p_/*Q*_s_ ratio ≥ 2. The mean NT-proBNP level was 84.4 ± 97.6 pg/ml. Notably, patients with a *Q*_p_/*Q*_s_ ratio ≥ 2 had significantly higher NT-proBNP levels (*P* < 0.001), higher PAm (*P* < 0.001), longer fluoroscopy time (*P* = 0.003), and longer procedure time (*P* < 0.001) compared to those with a *Q*_p_/*Q*_s_ ratio < 2.

**Table 1:** Baseline characteristics of children with ASD.

Variables	Total (*N* = 464)	*Q*_p_/*Q*_s_ < 2 (*N* = 334)	*Q*_p_/*Q*_s_ ≥ 2 (*N* = 130)	*P*-value
Male (*N*, %)	200 (43.1%)	143 (42.8%)	57 (43.8%)	0.840
Age (years)	7.81 ± 4.67	7.91 ± 4.15	7.55 ± 5.80	0.519
Body weight (kg)	27.4 ± 16.6	28.1 ± 15.9	25.5 ± 18.3	0.151
*Q*_p_/*Q*_s_	1.89 ± 0.67	1.56 ± 0.17	2.74 ± 0.72	<0.001
NT-proBNP (pg/ml)	84.4 ± 97.6	57.7 ± 49.9	152.9 ± 145.5	<0.001
Right atrium mean pressure	5.3 ± 3.3	5.0 ± 3.3	6.2 ± 3.0	<0.001
Pulmonary artery mean pressure	17.3 ± 4.9	16.6 ± 4.7	19.3 ± 4.8	<0.001
Fluoroscopy time (min)	14.4 ± 9.4	13.5 ± 8.8	16.7 ± 10.6	0.003
Procedure time (min)	44.5 ± 23.9	40.1 ± 20.2	55.6 ± 28.6	<0.001

ASD, atrial septal defect; NT-proBNP, N-terminal pro-B-type natriuretic peptide; *Q*_p_/*Q*_s_, pulmonary to systemic flow ratio.

**Figure 1:**
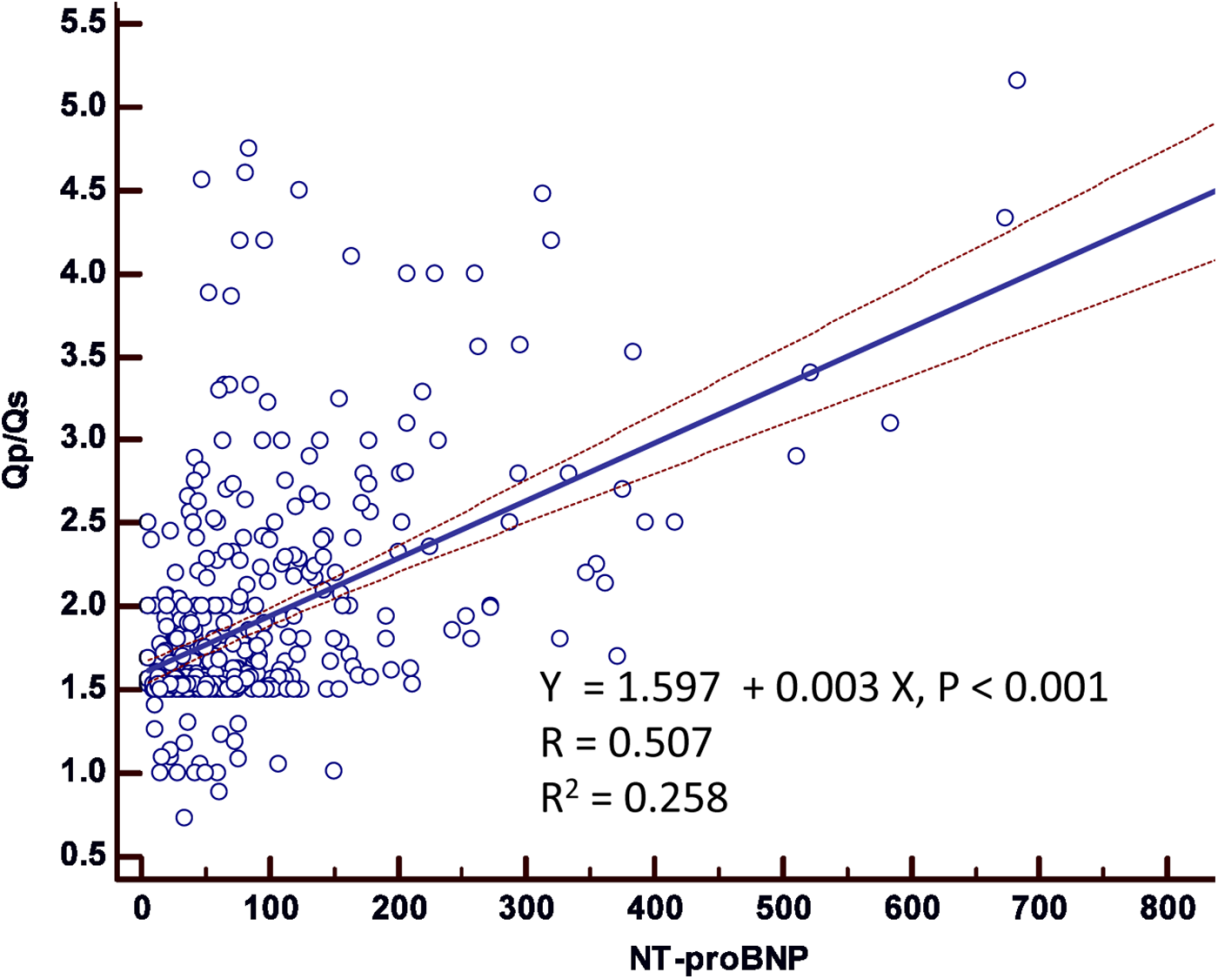
Scatter plot of association between NT-proBNP and *Q*_p_/*Q*_s_.

**Figure 2:**
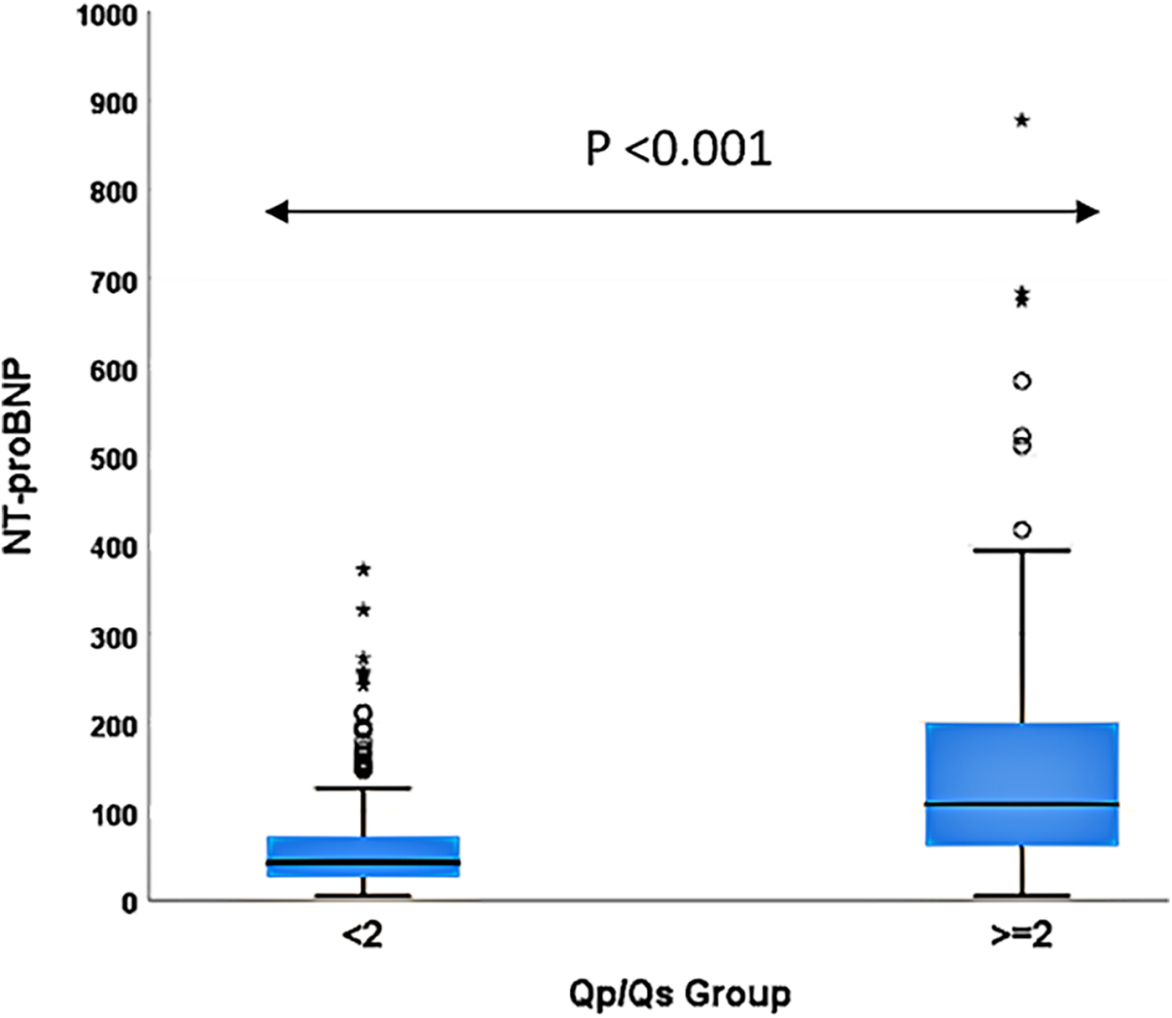
NT-proBNP levels according to *Q*_p_/*Q*_s_ groups.

Figure 1 illustrates the scatter plot of the association between NT-proBNP and *Q*_p_/*Q*_s_, demonstrating a significant positive correlation (*R* = 0.507, *P* < 0.001) with an *R*^2^ of 0.258. The linear regression model estimated that for each unit increase in NT-proBNP, there was a corresponding increase in *Q*_p_/*Q*_s_ by 0.003 units (*P* < 0.001). This indicates a moderate association between higher NT-proBNP levels and increased *Q*_p_/*Q*_s_ ratios. Figure 2 depicts the NT-proBNP levels stratified by *Q*_p_/*Q*_s_ group (<2 vs. ≥2). Patients with a *Q*_p_/*Q*_s_ ratio ≥ 2 exhibited significantly higher NT-proBNP levels compared to those with a *Q*_p_/*Q*_s_ ratio < 2 (*P* < 0.001). The box plot shows the distribution of NT-proBNP levels within each group, highlighting the greater dispersion and higher median levels in the *Q*_p_/*Q*_s_ ≥ 2 group.

Univariable analysis (Table 2) revealed a significant association between the *Q*_p_/*Q*_s_ ratio and NT-proBNP levels. For continuous *Q*_p_/*Q*_s_, the analysis showed that for each unit increase in *Q*_p_/*Q*_s_, NT-proBNP levels increased by 74.4 pg/ml (95% CI: 62.8–86.0, *P* < 0.001). Those with a *Q*_p_/*Q*_s_ ratio ≥ 2 showed a significant increase in NT-proBNP levels (Beta: 95.3, 95% CI: 69.8–120.7, *P* < 0.001). Multivariable analysis, adjusted for age, sex, body surface area, RAm, PAm, fluoroscopy time, and procedure time, confirmed that a higher *Q*_p_/*Q*_s_ ratio remained independently associated with elevated NT-proBNP levels. Specifically, the multivariable model indicated that for each unit increase in *Q*_p_/*Q*_s_, NT-proBNP levels increased by 68.3 pg/ml (95% CI: 47.0–89.6, *P* < 0.001). Furthermore, in the multivariable model, patients with a *Q*_p_/*Q*_s_ ratio ≥ 2 continued to show significantly higher NT-proBNP levels compared to those with *Q*_p_/*Q*_s_ < 2 (Beta: 72.7, 95% CI: 50.8–94.5, *P* < 0.001).

**Table 2:** Effect of *Q*_p_/*Q*_s_ groups on NT-proBNP levels after multivariable adjustment.

Variables	Uni-variable analysis			Multi-variable analysis^a^		
	Beta	95% CI	*P*-value	Beta	95% CI	*P*-value
*Q*_p_/*Q*_s_	74.4	62.8–86.0	<0.001	68.3	47.0–89.6	<0.001
*Q*_p_/*Q*_s_ group:						
<2	Reference			Reference		
≥2	95.3	69.8–120.7	<0.001	72.7	50.8–94.5	<0.001

NT-proBNP, N-terminal pro-B-type natriuretic peptide; *Q*_p_/*Q*_s_, pulmonary to systemic flow ratio.

^a^ Multi-variable model was adjusted for: age, sex, body surface area, right atrium mean pressure, pulmonary artery mean pressure, fluoroscopy time, procedure time.

Further analysis presented in Table 3 examined the effect of NT-proBNP levels on *Q*_p_/*Q*_s_ ratios. Univariable analysis indicated that continuous NT-proBNP levels were significantly associated with *Q*_p_/*Q*_s_, with a Beta of 0.003 (95% CI: 0.002–0.004, *P* < 0.001). Stratifying NT-proBNP into groups using <125 pg/ml as the reference, patients with NT-proBNP levels ≥125 pg/ml exhibited significantly higher *Q*_p_/*Q*_s_ ratios (Beta: 0.755, 95% CI: 0.567–0.943, *P* < 0.001). The multivariable analysis, adjusted for the same covariates, corroborated these findings, showing that NT-proBNP levels ≥125 pg/ml remained significantly associated with higher *Q*_p_/*Q*_s_ ratios (Beta: 0.664, 95% CI: 0.470–0.858, *P* < 0.001). Our study revealed significant differences in NT-ProBNP level between the *Q*_p_/*Q*_s_ < 2 and *Q*_p_/*Q*_s_ ≥ 2 groups (57.7 ± 49.9 vs. 152.9 ± 145.5 pg/ml; *P* < 0.001). The scatter plot of the association between NT-proBNP and *Q*_p_/*Q*_s_ showed a significant correlation (*P* = 0.001).

**Table 3:** Effect of NT-proBNP groups on *Q*_p_/*Q*_s_ levels after multivariable adjustment.

Variables	Uni-variable analysis			Multi-variable analysis^a^		
	Beta	95% CI	*P*-value	Beta	95% CI	*P*-value
NT-proBNP	0.003	0.002–0.004	<0.001	0.003	0.002–0.004	<0.001
NT-proBNP group:						
<125	Reference			Reference		
≥125	0.755	0.567–0.943	<0.001	0.664	0.470–0.858	<0.001

NT-proBNP, N-terminal pro-B-type natriuretic peptide; *Q*_p_/*Q*_s_, pulmonary to systemic flow ratio.

^a^ Multi-variable model was adjusted for: age, sex, body surface area, right atrium mean pressure, pulmonary artery mean pressure, fluoroscopy time, procedure time.

## Discussion

This study represents the largest cohort to date examining the strong relationship between non-invasive NT-proBNP levels and invasive *Q*_p_/*Q*_s_ measurements in children with ASD. Smaller-scale studies with limited sample sizes have reported that NT-proBNP levels were higher in their ASD group (79 pg/ml) than in the control cohort (57 pg/ml), with statistical significance (*P* < 0.05). This finding highlights the potential of NT-proBNP level to serve as a diagnostic indicator for ASD size, aligning with echocardiographic assessments (15). Additionally, a prior study found elevated serum NT-proBNP levels in individuals with larger defects. These studies indicate that employing NT-proBNP level as a diagnostic marker can effectively anticipate the size of these cardiac defects (16).

The persistent left-to-right shunt in heart defects continuously influences the pulmonary artery, inducing vascular remodeling. This process leads to a progressive increase in arterial pressure and resistance, ultimately resulting in pulmonary hypertension. Numerous humoral regulators actively participate in the intricate regulation of the cardiovascular system during the progression of this condition. The levels of circulating NTproBNP have been shown to be correlated with pulmonary hypertension. NTproBNP levels have also been correlated with mean pulmonary pressure, pulmonary vascular resistance, right atrial pressure, and cardiac index (17, 18). Elevated NTproBNP levels might indicate remodeling of the right ventricle, resulting in compromised systolic function of the right ventricle (19). We observed a positive correlation between NTproBNP increase and shunt volume, as measured by cardiac catheterization, in patients with ASD. NTproBNP demonstrated acceptable accuracy in predicting intracardiac shunt magnitude in ASD cases. A previous study with a smaller sample size demonstrated an association between B-type natriuretic peptide (BNP) levels and shunt severity in septal defect patients, indicating a significant positive correlation between plasma BNP levels and the magnitude of the shunts (20). Combining NTproBNP with Doppler echocardiography enhances prognostic accuracy, sensitivity, specificity, and predictive values for pulmonary hypertension in CHD patients. These outcomes align with the findings of Yin et al. that showed a combined assessment of NTproBNP/BNP and doppler echocardiography enhances diagnostic value and aids in clinical decision-making (21, 22). The combination of Doppler echocardiography with NTproBNP provides enhanced diagnostic efficacy for pulmonary artery hypertension associated with CHD. This is particularly notable when Doppler echocardiography yields negative results in screening for pulmonary arterial hypertension in patients (23–26). Holmstrom et al. and Choi et al. have suggested that consecutive BNP measurements can offer clinically relevant insights, and may be useful in the assessment of shunt severity as well as approach to managing preterm infants diagnosed with a patent ductus arteriosus (27, 28). BNP determinations could aid in the identification of children with septal defects complicated by pulmonary hypertension (29).

However, there are some limitations in this study. First, neither *Q*_p_/*Q*_s_ nor NT-proBNP could accurately predict the size of the ASD. This may be due to measurement errors as well as the association between larger ASD size and increased pulmonary pressure. Second, for clinical convenience, we did not adjust NT-proBNP levels based on age, which could introduce bias. Lastly, this study is a retrospective cohort study that have several limitations, including reliance on potentially incomplete or inaccurate data, difficulty in controlling for confounding factors, selection bias due to the non-random selection of participants, and challenges in establishing clear causality due to time errors. These limitations necessitate careful interpretation of the study's findings to ensure reliability and validity. Large multi-institutional studies will need to be conducted to conclusively determine the clinical value of NT-proBNP as a biomarker for shunt severity in pediatric patients with ASD. In conclusion, our study demonstrates a correlation between non-invasive NT-proBNP levels and invasive *Q*_p_/*Q*_s_ measurements in children with ASD.

## Data Availability

The raw data supporting the conclusions of this article will be made available by the authors, without undue reservation.
